# P66Shc and vascular endothelial function

**DOI:** 10.1042/BSR20182134

**Published:** 2019-04-30

**Authors:** Santosh Kumar

**Affiliations:** Department of Internal Medicine, Carver College of Medicine, University of Iowa, Iowa City, IA, U.S.A.

**Keywords:** endothelial function, oxidative stress, p66Shc

## Abstract

Dysfunctional endothelium is an early change in vasculature known to be associated with atherosclerosis. Among many regulators of vascular endothelial function, p66Shc has consistently been shown to mediate endothelial dysfunction. Over more than three decades of active research in the field of the physiological function of p66Shc, regulation of vascular endothelial functions has emerged as one of the most robust effects in a broad range of pathological conditions including hyperlipidemia, diabetes, and aging. A significant understanding has been developed with respect to the molecular signaling regulating the oxidative function of p66Shc in endothelial cells and its targets and regulators. In addition, novel regulatory modifications of p66Shc controlling its oxidative function, subcellular distribution, and stability have also been reported. This review will focus on summarizing the molecular signaling regulating the oxidative function of p66Shc and its role in vascular endothelium.

## The discovery of p66Shc

**Figure 1:**
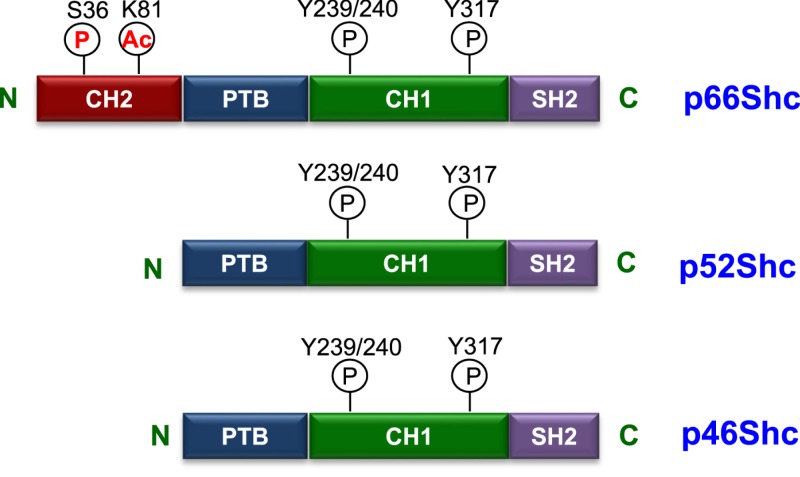
Modular structure of Shc proteins All the three isoforms of Shc share the phosphotyrosine binding (PTB), CH1, and Src2 homology domain. The CH1 domain gets tyrosine phosphorylated (Y-239, 240, and 317) in response to activation of receptor tyrosine kinase. In the CH2 domain of p66Shc, phosphorylated on Ser36 and acetylation of Lys81 (K81) drives the oxidative function of p66Shc. Abbreviations: Ac, acetylation; P, phosphorylation.

Proteins having Src homology 2 (SH2) domains participate in cytoplasmic signaling in response to the stimulation of receptor tyrosine kinases, e.g. growth factor receptors [1]. This important role of SH2 domain-containing proteins in facilitating the growth receptor signaling led to the discovery of Shc. In mice, transgenic overexpression of Shc proteins induced tumor formation supporting the role of Shc proteins in the activation of mitogenic pathways. Subsequent investigation into the transcriptional control of *Shc* gene identified the presence of two different initiation codons, which suggested the possibility of two transcripts of estimated molecular weights of 52 and 46 kDa. Surprisingly, immunoblotting with Shc antibody revealed the presence of an additional protein band at a higher molecular weight (66 kDa) indicating that the two initiation codons produce three different isoforms, p46, p52, and p66Shc. In 1997, Migliaccio et al. [2] reported that p66Shc and p52/p46Shc are actually two different transcripts starting from the same locus but p66Shc is transcribed from exon2-13 while p52/46Shc are transcribed from exon1, part of exon2(2a) and exon 3-13(2). All three Shc isoforms share a common phospho-tyrosine binding domain (PBD) at N-terminus, a collagen homology 1 (CH1) domain in the center, and a SH2 domain present at C-terminus (Figure 1). In addition to the PBD, CH1, and SH2 domains, p66Shc has an extra collagen homology 2 (CH2) domain at the N-terminus which is sufficient to replicate the function of p66Shc [2].

**Figure 2:**
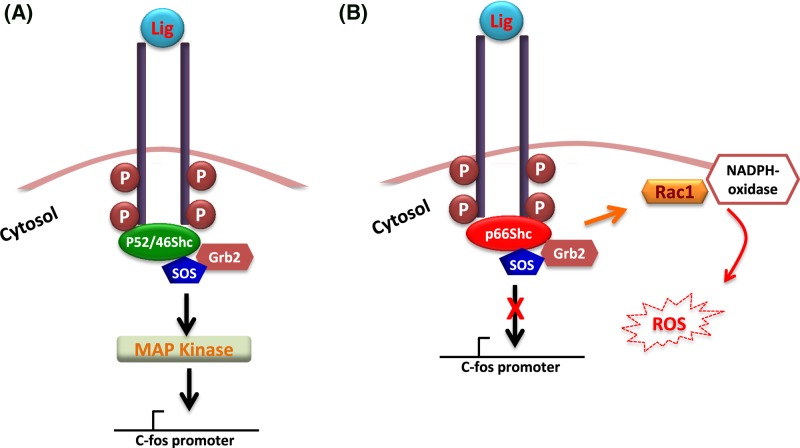
Differential signaling of p52/46Shc and p66Shc Following stimulation with ligand, the cytoplasmic tyrosine kinase phosphorylates and recruits Shc proteins to form a complex with Grb2 and Sos which subsequently activates the MAP kinase signaling (**A**). When p66Shc participates in the complex formation it activates Rac1 not the RAS (**B**). Abbreviation: Lig, ligand.

Activation of epidermal growth factor receptor (EGFR, a type of receptor tyrosine kinase) recruits Shc adaptor proteins at the cytoplasmic tail which forms a stable complex with Grb2 (another adaptor protein). This activates signal transduction via mitogen activated protein (MAP)-kinase which controls cellular growth and differentiation. Interestingly, even though p66Shc is phosphorylated in response to the EGFR activation it failed to transmit the EGFR activation signal to the MAP-kinase. In contrast, p66Shc inhibited the *c-fos* promoter activity (Figure 2). Thus, based on their ability to initiate MAP-kinase signaling and activate the *c-fos* promoter (a growth factor-regulated gene), p66Shc was segregated from p52Shc and p46Shc as a redox protein.

In an attempt to determine the cellular function of p66Shc, mouse embryonic fibroblasts (MEFs) were treated with either EGF to activate canonical tyrosine kinase signaling or were exposed to H_2_O_2_ and UV irradiation to induce cellular stress. Unlike EGF treated MEFs, exposure to the apoptotic stimulus reduced the electrophoretic mobility of p66Shc suggesting the post-translational modifications of p66Shc. This post-translational modification was identified as phosphorylation at Ser36 in the CH2 domain of p66Shc [3]. The finding that the signal for oxidative damage is mediated via p66Shc was further supported by the better tolerance of p66Shc^−/−^ mice to paraquat challenge and their increased lifespan. Thus, a member of the growth factor receptor family was identified as promoting cellular oxidative stress and organismal longevity. Although the recognition of p66Shc as a ‘longevity protein’ is ambiguous [3–5] the oxidative function of p66Shc in vascular health is unequivocal [6] and is the focus of this review.

## Regulation of p66Shc function

The defensive phenotype of p66Shc^−/−^ mice against a variety of challenges established the pathological role of p66Shc. In subsequent mechanistic studies, the existence of multiple levels of regulatory controls on the oxidative function of p66Shc were identified. In pathological conditions, there is dysregulation in transcriptional control of p66Shc expression and activation of the oxidative function of p66Shc. We will discuss the molecular signaling involved in both transcriptional and post-translational control of p66Shc.

### The transcriptional control of p66Shc expression

**Figure 3:**
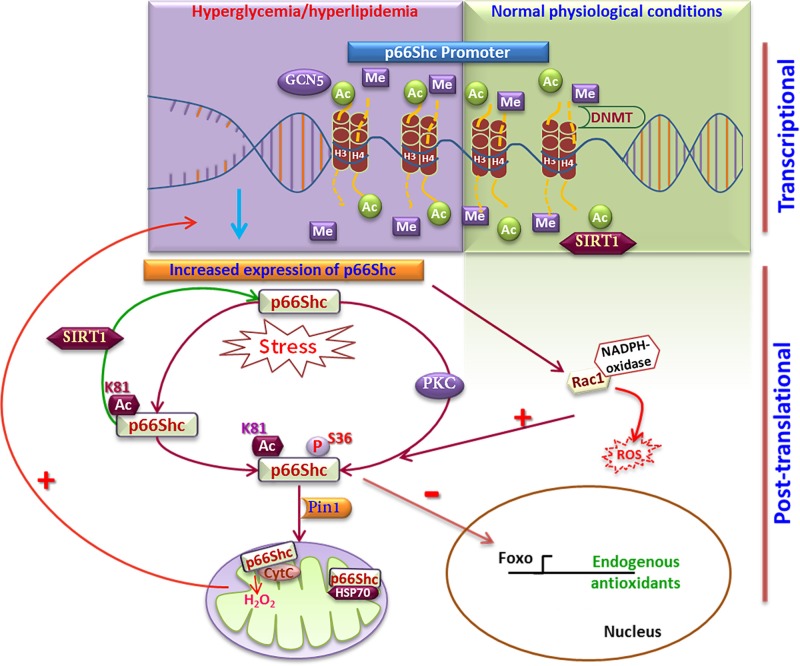
Cellular regulation of p66Shc In physiological conditions, p66Shc transcription is reduced due to a compact chromatin structure in the promoter region. In pathological conditions like metabolic dysfunction, epigenetic chromatin modifications (e.g. reduced methylation and increased acetylation) in the promoter region of p66Shc lead to increased transcription of p66Shc. Further, p66Shc is acetylated and phosphorylated leading to its translocation to the mitochondria and ROS production. In the nucleus, p66Shc interferes with Foxo-dependent transcription of endogenous antioxidants. Abbreviations: Ac, acetylation; CytC, cytochrome *c*; DNMT, DNA methyltransferase; GCN5, acetyl transferase; H3 and 4, Histone 3 and 4; Me, methylation, P, phosphorylation; PKC, protein kinase C; ROS, reactive oxygen species.

The protein expression of p66Shc varies among tissues [7]. This difference in the level of p66Shc expression among different tissues/organs indicated that a transcriptional/post-translational control system exists which governs the levels of p66Shc. However, whether the organ/tissue-specific difference in the expression of p66Shc is due to the short lifespan of p66Shc protein or due to the transcriptional repression of p66Shc was not known. The use of alternate promoters by the different isoforms of Shc as well as an additional start codon for p66Shc transcription (present in the exon 2) had previously been reported [2]. The simultaneous absence of transcript as well as protein and the restitution of p66Shc expression by transient transfection of p66Shc constructs in cells not expressing endogenous p66Shc, provided the clue for possible transcriptional repression of the p66Shc promoter by specific transcription factor/s in a cell-specific manner [8]. Both deacetylase inhibitors and demethylating agents increased p66Shc protein expression in a dose-dependent manner, indicating the epigenetic regulation of p66Shc promoter via histone deacetylation and cytosine methylation (Figure 3). This was the first study to report that a histone deacetylase or methyltransferase could regulate p66Shc transcription. Subsequently, Kim et al. (2012) [9] reported the methylation of the p66Shc promoter by low-density lipoprotein (LDL) thereby increasing its transcription. Importantly, the methylation noted in the p66Shc promoter occurs on a CpG site (regions of DNA where a cytosine nucleotide is followed by a guanine nucleotide in the linear sequence of bases) which is not a classical CpG island.

The existence of another independent transcriptional regulation of p66Shc originated from the finding that p53 up-regulates the expression of p66Shc and p66Shc is indispensable for p53-induced apoptosis [10]. The tumor repressor p53 is an established transcriptional regulator and is known to be associated with advanced vascular pathologies [11]. Therefore, the possible transcriptional control of p66Shc by p53 was investigated. Chromatin immunoprecipitation assay identified a 22–24 bp length of putative p53 response element bound to the p66Shc promoter [12]. Importantly, the identification of this p53 response element also redefined the limit of the p66Shc promoter region.

Later, Zhou et al. (2011) [13] discovered that SIRT1 (a class III histone deacetylase) directly binds to the p66Shc promoter (−508 to −250 bp) and represses p66Shc transcription by deacetylating Lys9 in histone H3. It was reported that the mRNA expression of p66Shc increases in response to both high glucose and oxidized-LDL (o-LDL) in HUVECs (human umbilical vein endothelial cells) and this increased expression of p66Shc is inhibited by the overexpression of SIRT1. In addition, in the setting of diabetes, the endothelium-specific transgenic overexpression of SIRT1 in mice reduced p66Shc expression at both the mRNA and protein levels compared with wild-type diabetic mice.

Paneni et al. (2012) [14] next reported that hyperglycemia places a transcriptional memory on p66Shc promoter which drives the vascular endothelial dysfunction in diabetes. In this study, it was reported that hyperglycemia is associated with hypomethylation of the p66Shc promoter as well as increased expression of GCN5 (a histone acetyl transferase) and p66Shc. Interestingly, normalization of blood glucose failed to lower the p66Shc expression level. However, the hyperglycemia-associated increase in p66Shc expression did respond to changes in blood glucose levels following the knockdown or pharmacological inhibition of GCN5, indicating that the hyperglycemia-induced epigenetic regulation of p66Shc transcription is initiated by the increase in blood glucose level but continues independently due to these epigenetic modifications. A recent report by Costantino et al. (2017) [15] showed that the methyltransferase SUV39H1 recruits JMJD2C (demethylase) and SRC-1 (acetyltransferase) to the p66Shc promoter leading to the repression of p66Shc transcription. This is in accordance with the known down-regulation of SUV39H1 in obesity and increased expression of p66Shc. Interestingly, it is known that SUV39H1 is a target of SIRT1 and SIRT1 increases the stability of SUV39H1 by preventing its degradation via polyubiquitination [16]. However, whether SIRT1-dependent transcriptional repression of p66Shc is also influenced by SIRT1-mediated increase in SUV39H1 is not known.

Nuclear factor erythroid 2-related factor 2 (Nrf2)-dependent antioxidant response element (ARE) activates the transcription of an array of antioxidant genes under oxidative stress conditions [17]. In the setting of cancer, Nrf2 binds to the demethylated promoter region of p66Shc and thereby promotes its transcription [18]. This upregulation is supported by another study identifying a specific Nrf2-binding site in the p66Shc promoter and increased p66Shc expression upon activation of Nrf2 [19]. This study also reported that activation of the Nrf2 pathway by hemin increased S36 phosphorylation of p66Shc but did not lead to the mitochondrial translocation noted by others in different cell types. These findings indicate two very important possibilities in p66Shc signaling: (i) the transcriptional control and the cellular signaling of p66Shc is specific to cell type and (ii) the S36 phosphorylation of p66Shc may have an additional function.

### The molecular signaling of p66Shc

In general, the Shc family of proteins has a phosphotyrosine binding (PTB) domain at the N-terminus and an SH2-domain at the C-terminal end. The CH1 domain present between the PTB and SH2, which is common among p66Shc/p52/p46, holds the functional cue for signal transduction by Shc. Phosphorylation in this domain is essential for Shc–Grb2–Sos complex formation and subsequent MAP-kinase signaling [20]. Similar to the functional regulation of Shc proteins by its CH1 domain, the Ser36 phosphorylation in the CH2 domain regulates the oxidative function of p66Shc [3].

It is widely accepted that protein kinase C (PKC) β (PKC-β) regulates the S36 phosphorylation of p66Shc [21], although other kinases have also been reported to promote the S36 phosphorylation [22,23]. A study by Shi et al. (2011) [22] reported that o-LDL increases the phosphorylation of PKC-β2 as well as c-Jun N-terminal kinase (JNK) in human aortic endothelial cells (HAECs). Both of these enzymes can phosphorylate p66Shc at S36, thereby promoting reactive oxygen species (ROS) production. This S36 phosphorylation marks p66Shc for recognition by the prolyl isomerase PIN1 and p66Shc is subsequently dephosphorylated before its translocation to the mitochondria [21]. In the mitochondria, p66Shc is localized in the inter-mitochondrial space as an inhibitory complex with mtHSP70 [24]. This complex dissociates in response to external stimuli and oxidizes cytochrome *c* generating hydrogen peroxide [24]. Exposure to UV radiation leads to the opening of the mitochondrial permeability transition pore along with activation of caspase-3 and the release of cytochrome *c*. The mechanism by which p66Shc increases the mitochondrial ROS level was identified by electrochemical studies demonstrating that p66Shc is capable of electron transfer and it interacts with cytochrome *c* via the cytochrome *c* binding (CBP) domain present at the junction of the CH2-PTB domain [25].

Regulation of the oxidative function of p66Shc by Ser36 phosphorylation and subsequent mitochondrial translocation is the most widely investigated post-translational modification of p66Shc. In addition to the increase in mitochondrial ROS production by p66Shc, Rac1 mediated activation of NADPH-oxidase is another mechanism for p66Shc-induced oxidative stress. Exploring the site of interaction for the binding of p66Shc to Grb2–Sos complex, it was reported that the PPLP motif in the proline-rich CH2 domain of p66Shc competes with Sos for the C-terminal SH3 domain of Grb2 and thereby promotes Rac1 activation [26]. In addition, Rac1 increases the stability of p66Shc via Ser54 and Thr386 phosphorylation thereby preventing the ubiquitination and degradation of p66Shc [20].

Other than phosphorylation in response to oxidant stress, oxidative modification in the CH2 domain was also reported to mediate the mitochondrial effect of p66Shc [27]. The significance of the CH2 domain in mediating the mitochondrial effect of p66Shc was studied in isolated mitochondria using purified recombinant CH2 domain containing a cytochrome *c* binding domain (CH2-CB). During elution of the recombinant CH2-CB fragment, two distinct peaks of oligomers with CH2-CB appeared. These oligomers were sensitive to the presence of reducing agents and were characterized as di- and tetramers of p66Shc which interact via Cys59. Interestingly, these purified oligomers were efficient in inducing ROS production and mitochondrial swelling. This was one of the earliest reports suggesting p66Shc as redox sensor and oxidant.

To this point, serine/threonine modifications were the only known post-translational modification of p66Shc regulating its function and stability. Knowing the opposing function of p66Shc and SIRT1 in many pathophysiologies, including vascular endothelial dysfunction, we investigated whether lysines present in the CH2 domain of p66Shc are targeted by the SIRT1 deacetylase. Interestingly, the acetylation of Lys81 appeared to be imperative for the oxidative function of p66Shc [28]. We showed that SIRT1 directly interacts with p66Shc and deacetylates Lys81 in the CH2 domain. The Lys81 in p66Shc was hyperacetylated both in diabetic conditions and following knockdown of SIRT1. Preventing p66ShcK81 acetylation (by mutation to p66ShcK81R) inhibited the hyperglycemia-induced S36 phosphorylation and subsequent p66Shc mitochondrial translocation and ROS production. This was the very first *in vivo* study demonstrating the importance of a post-translational modification of endogenous p66Shc in regulating vascular endothelial health. Surprisingly, the dependence of S36 phosphorylation on p66ShcK81-acetylation was absent when we stimulated the HUVECs with vascular endothelial growth factor (VEGF), suggesting the stimulus-specific role of p66ShcK81 acetylation.

### Molecular targets of p66Shc

Migliaccio et al. (1999) [3] demonstrated that p66Shc regulates organismal lifespan. This report recognized p66Shc as a promising candidate to control aging and cellular lifespan. Subsequently, molecules known to be involved in the regulation of cell death and organismal lifespan were investigated for their relationship with p66Shc. In 2002, two separate groups of researchers reported the interaction of p66Shc with two very important proteins, Foxo and p53, which were known to regulate many cellular processes controlling cellular longevity. The study by Nemoto and Finkel (2002) [29] demonstrated that the oxidant-induced inhibition of Foxo activity is mediated via p66Shc. Increased activity of Foxo increases lifespan and oxidants inhibited the activity of FKHRL1 (a Foxo protein) in a p66Shc-dependent manner. MEFs carrying a deletion of p66Shc showed increased activity of FKHRL1, which increased the transcription of catalase. This study demonstrated that the ROS produced by p66Shc diminishes the endogenous antioxidant defense system.

Trinei et al. (2002) [10] reported that p66Shc mediates the apoptotic signal of the tumor suppressor p53. Deletion of p66Shc prevented the p53-induced increase in cellular apoptosis, cytochrome *c* release, and ROS production. Using tissues from p66Shc^−/−^ mice, it was demonstrated that the redox function of p66Shc positively correlates with the expression level of p53. In endothelial cells, p66Shc also facilitates the transcriptional repression of Kruppel-like factor-2 (KLF2) transcription factor, a key regulator of endothelial function [30]. P66Shc impairs the binding of myocyte enhancing factor-2A (MEF2A) in the core KLF2 promoter and thereby down-regulates KLF2 expression and represses the expression of the vasoprotective target gene *thrombomodulin*.

Another important function of p66Shc in mediating intracellular signaling is via regulation of endothelin1 in primary human mesangial cells. Chahdi and Sorokin (2008) [31], showed that in response to stimulation with endothelin1, p66Shc functions as an adaptor for FOXO3a and β-pix and forms a complex that promotes the cell proliferation via an ERK-dependent pathway.

In cardiomyocytes, p66Shc mediates the α1-adrenergic stimulation-induced inactivation of FOXO3a transcription factors by AKT-dependent phosphorylation [32] which down-regulates endogenous antioxidants. Interestingly, p66Shc did not prevent the hypertrophy of cardiomyocytes in response to adrenergic stimulation which is contrary to the common understanding that p66Shc does not particpiate in growth receptor signaling. This suggests the tissue and stimulus-specific role of p66Shc in regulating cellular fate or function.

These evidences support the existing notion that p66Shc is an important regulator of cellular function. Most of the effects of p66Shc are initiated via its redox function, however the spectrum of effects ranges from the regulation of the cytoplasmic signaling to transcriptional control. Although many more parallel studies appeared with respect to the role of p66Shc in pathologies like myocardial injury [33], nephropathy [34], platelet activation [35], and atherosclerosis [36], here I have specifically focused on the role of p66Shc in molecular signaling regulating vascular endothelial function.

## p66Shc in vascular physiology

Among many factors regulating the function of the circulatory system, the innate property of blood vessels to control their diameter plays a central role. Endothelial cells, which are the first and foremost cell layer segregating the body from circulating factors, also control the diameter of the blood vessel lumen [37]. The inability of endothelium to facilitate the relaxation of vascular smooth muscle has been noted in many pathological conditions and has been regarded as an early pathological change in the vasculature [38]. Release of nitric oxide (NO), endothelium derived relaxing factor (EDRF), by endothelial cells relaxes vascular smooth muscle via activation of cGMP. The impaired function of vascular endothelium to produce EDRF has been known to promote the vascular pathologies like, atherosclerosis and stroke.

The redox protein p66Shc is expressed in endothelial cells and its expression changes with alteration of metabolic health. The ample expression of p66Shc in vascular endothelium and numerous studies showing the *in vivo* effect of p66Shc on vascular function has established p66Shc as a prominent therapeutic target for vascular dysfunction. Many studies have reported the importance of p66Shc in endothelial function with respect to a wide variety of stimuli and pathologies. These studies also discovered many novel mechanisms mediating and governing the effect of p66Shc on vascular endothelium. A comprehensive discussion of how p66Shc contributes to vascular endothelial dysfunction in different pathologies is presented here.

### Aging-induced endothelial dysfunction and p66Shc

With age, impaired endothelial function has been reported in both animals and humans [39–41]. Aging impairs the endothelial production of NO [42]. The p66Shc^−/−^ mice are resistant to oxidative stress and have increased lifespan [3] and both of these are known to contribute to vascular endothelial dysfunction [39,43]. Evaluating the vascular endothelial function in the aged p66Shc^−/−^ mice showed that these mice are protected against the aging-induced endothelial dysfunction, increase in inducible-NO (iNOS), superoxide production and reduction in NO bioavailability [44]. This was the first study suggesting a link between p66Shc and vascular endothelial function in aged mice.

A year later, Yamamori et al. (2005) [45] reported that p66Shc impairs endothelial NO production. Knocking down p66Shc increased the Ser1177 phosphorylation of endothelial NO synthase (eNOS), which is critical for NO production by eNOS. A later study corroborated these findings in a different vascular bed. The basilar arteries and the femoral arteries from the aged p66Shc^−/−^ mice were evaluated for aging-induced endothelial dysfunction. The p66Shc^−/−^ mice were protected against age-associated endothelial dysfunction in basilar arteries [46]. The femoral arteries did not show age-induced changes in endothelial function. The finding from these two studies that p66Shc regulates ROS and NO level in vascular endothelium set the stage for many future studies examining the role of p66Shc in different pathophysiological conditions.

### P66Shc in diabetic endothelial dysfunction

The contribution of p66Shc to diabetic endothelial dysfunction has been studied in great detail. The first study reporting an association between p66Shc and diabetes showed that in patients with type II diabetes, there is an increased expression of p66Shc in circulating peripheral blood mononuclear cells [47]. Interestingly, among many tissues investigated for the expression of p66Shc, hematopoietic cells are reported to have an undetectable level of p66Shc [7]. A year later, two independent groups of investigators reported that p66Shc^−/−^ mice are protected against streptozotocin-induced diabetic cardiomyopathy and glomerulopathy [48,49]. Subsequently, Camici et al. (2007) [50] reported that diabetes increases the expression of p66Shc in the vasculature and p66Shc knockout mice are protected against diabetes-induced vascular endothelial dysfunction and oxidative stress. The p66Shc knockout mice showed a reduced level of nitrotyrosine expression and lipid peroxidation along with preserved NO bioavailability. However, p66Shc^−/−^ mice were not protected against the induction of diabetes or the diabetes-induced increase in glycosylated hemoglobin, endothelial NO synthase, or total cholesterol.

Different molecular and signaling pathways have been evaluated for their contribution in the activation of p66Shc. In diabetes, glucose modified products called advanced glycation end products (AGEs) accumulate. In response to AGE, the receptor for AGEs (RAGE) promotes oxidative stress and inflammation [51] and stimulation of the advanced glycation end receptor 1 (AGER1, a variant of RAGE) reduces AGE levels and suppresses oxidative stress, inflammation, and activation of RAGE [52]. In an early study, it was reported that p66Shc^−/−^ mice are protected against AGE-induced renal damage and circulating isoprostane 8-epi-PGF2-α (a marker of oxidative stress) and renal oxidative stress [53]. Later, Cai et al. (2008) [54] reported that Ser36 phosphorylation of p66Shc mediates the AGE-induced oxidative stress. In HEK-293 cells, AGE increased the p66Shc-S36 phosphorylation, inactivated the Foxo transcription factor (FKHRL1), and suppressed the endogenous antioxidant level. In smooth muscle cells, hyperglycemia increased the expression of p66Shc which impaired the IGF-1 stimulated phosphoinositide (PI)-3 kinase, AKT activation, and subsequent cell survival. Knockdown of p66Shc restored the IGF1-stimulated AKT activation [55].

Several other studies also focused on the alterations in transcriptional control of p66Shc in diabetes. Changes to epigenetic modifications on the p66Shc promoter, like acetylation and methylation were noted to be associated with diabetes, which has recently been reviewed [56]. Although many proteins have been implicated in the regulation of p66Shc transcription, SIRT1 is the only one which has been shown to directly bind to the p66Shc promoter (−508 to −250 bp), which represses p66Shc transcription by deacetylating the histone [13]. This explains the observed phenomena of reduced expression of SIRT1 and increased expression of p66Shc in endothelial cells due to diabetes. In addition, another study reported that GCN5, an acetyl transferase, mediates the hyperglycemia-induced increase in p66Shc expression and this increase in expression of p66Shc is normalized with reduction in blood glucose suggesting the formation of a ‘vascular hyperglycemic memory’ for p66Shc [14]. Interestingly, this hyperglycemic memory fades away in the presence of antioxidants, suggesting that oxidative stress is the driving force for p66Shc transcription in hyperglycemia.

The inverse relationship between p66Shc and SIRT1 expression was known in many pathologies including aging, dyslipidemia, and vascular dysfunction. We took a different approach to investigate the possible interaction between p66Shc and SIRT1 [28]. Knowing the fact that in addition to the nuclear proteins like histones, SIRT1 also targets many cytosolic proteins for deacetylation, we asked whether p66Shc is a direct target of SIRT1 deacetylase. However, the p66Shc acetylation was not known. We reported that p66Shc is acetylated at Lys81 (in the CH2 domain) by p300 (acetyl transferase) and is deacetylated by SIRT1. The p66Shc acetylation at Lys81 is increased in hyperglycemia and is necessary for Ser36 phosphorylation, ROS production and mitochondrial localization of p66Shc. Interestingly, mice having endothelial specific transgenic overexpression of p66ShcK81R (an acetylation-deficient mutant of p66ShcK81) were protected against hyperglycemia-induced endothelial dysfunction and oxidative damage. In addition, mice carrying a CRISPR-induced mutation of endogenous p66Shc Lys81 (p66ShcK81R knockin mice) showed a similar phenotype supporting the concept that the K81 modification in p66Shc drives the oxidative function of p66Shc in the vasculature. Interestingly, these p66ShcK81R knockin mice were also protected against diabetic down-regulation of vascular SIRT1. This corroborates the finding of our earlier study showing that ROS produced by p66Shc down-regulates the level of SIRT1 via up-regulation of miR-34a [57]. This is the first study discovering a novel post-translational modification and validating the same *in vivo* in endogenous p66Shc.

### P66Shc in dyslipidemia-induced vascular dysfunction

Impaired endothelial function was first demonstrated in patients with coronary artery disease (CAD) [38] and hyperlipidemia is one of the key factors promoting CAD [58–60]. Many clinical and experimental findings suggested the role of increased oxidative stress in hyperlipidemia-induced damage of vasculature structures [61–63]. Exploring the resistance of p66Shc^−/−^ mice to oxidative stress, Napoli et al. (2002) [64] evaluated the hyperlipidemia-induced systemic oxidative stress and damage to the vasculature in p66Shc^−/−^ mice. The p66Shc^−/−^ mice developed less aortic lesion, oxidative stress and apoptosis, however the serum lipid level in p66Shc^−/−^ mice was not different than wild-type. In a subsequent study using hypercholesterolemic apolipoprotein E (ApoE^−/−^) mice, it was shown that genetic deletion of p66Shc protects against atherosclerotic lesion formation [65]. Investigating the mechanism involved in the regulation of the redox function of p66Shc and hypercholesterolemia, it was reported that in endothelial cells, o-LDL initiates ROS production by NADPH-oxidase via engaging the o-LDL receptor-1. This promotes the phosphorylation and activation of PKC-β2 and JNK. Both the active PKC-β2 and JNK phosphorylate p66ShcS36 thereby further amplifying the ROS production [29]. Later, Kim et al. (2012) [9] reported that LDL up-regulates p66Shc transcription by inducing hypomethylation of the p66Shc promoter in endothelial cells which increases ROS production and induces endothelial dysfunction. In addition, we also reported that the oxidative function of p66Shc mediates the activation of β-catenin by Wnt3a in endothelial cells [66].

Another interesting mechanism for the endothelial effect of p66Shc has been proposed via the uncoupling of e-NOS. In pathological conditions, uncoupling of e-NOS promotes ROS production in endothelium [67]. Shi et al. (2014) [68] reported that in basal conditions, inhibiting eNOS function in endothelial cells increased p66ShcS36 phosphorylation. Interestingly, when stimulated with o-LDL, inhibition of eNOS function reduced p66ShcS36 phosphorylation. This paradoxical effect of eNOS on p66Shc phosphorylation suggests that eNOS produces NO which prevents the oxidative activation of p66Shc; however, when the eNOS is uncoupled, the ROS produced by eNOS further promotes p66ShcS36 phosphorylation and activates the vicious production of ROS in endothelium.

In clinical studies, increased expression of p66Shc was reported in peripheral blood monocytes (PBMs) of patients with CAD [69]. Interestingly, this increase was specifically associated with patients having coronary syndrome not with having stable CAD [70]. Further, the increased level of p66Shc in PBMs was negatively correlated with the endothelial function assessed by flow-mediated dilation [71]. These studies indicate the presence of a dynamic relationship between CAD and the level of p66Shc mRNA. Further studies are needed to establish the significance of this increase in p66Shc mRNA in PBMs.

### p66Shc and vascular dysfunction in hypertension

It was known that angiotensin II does not promote hypertension and endothelial dysfunction in p66ShcRNAi mice [12]. This indicated that p66Shc probably mediates the hypertension-induced pathological changes in the vasculature. The study by Spescha et al. (2014) [72] investigated whether mimicking hypertension-induced mechanical stretch affects the endothelial function via p66Shc. The study reported increased S36 phosphorylation of p66Shc in spontaneously hypertensive rats (SHR) and showed that the cyclic stretches of endothelial cells increase S36 phosphorylation of p66Shc and reduction in NO bioavailability. These studies suggested a prominent role of p66Shc in the vascular effect of hypertension. Miller et al. (2016) [73] evaluated the role of p66Shc in the rat model of genetic hypertension by using the Dahl salt-sensitive (SS) rats. They manipulated p66Shc expression by cross-breeding the SS rats with either p66Shc knockout rats or rats with a knockin of p66Shc non-phosphorylatable at S36 (p66ShcSer36Ala). Interestingly, the genetic deletion of p66Shc rescued the microvascular function in hypertensive rats, but the rats having p66ShcSer36Ala failed to replicate the p66Shc^−/−^ rats phenotype. Also, the p66ShcSer36Ala rats showed an impaired response to the stimulation with endothelin1. Interestingly, these p66ShcSer36Ala rats were protected against diabetic renal microvascular dysfunction [34]. The findings from this later study indicate that S36 phosphorylation is not important in mediating the effect of p66Shc on the vasculature in the setting of hypertension however, it may be important in mediating the diabetes-induced vascular dysfunction.

## Pharmacological manipulation of p66Shc

The above studies show that p66Shc is an important regulator of vascular endothelial function however the tools to regulate the p66Shc have not been well developed yet. The inhibitors of protein kinases (PKC-β, PKC-δ, JNK) and PIN1 which are responsible for the oxidative activation of p66Shc has been used in experimental studies to control the oxidative function of p66Shc. Unfortunately, the specific activator or inhibitor p66Shc function is still unavailable. The existence of closely related multiple isoforms of p66Shc, p52 and p46, as well as the lack of exclusive signaling mechanism for the p66Shc activation are some of the key hurdles in this process.

In conclusion, the role of p66Shc in regulating vascular function is well established. Knowledge of the molecular mechanisms regulating p66Shc function has also advanced in recent years. However, p66Shc function is differentially regulated depending on its distribution and concomitant pathophysiological conditions. Therefore, more *in vivo* studies are needed to support the known molecular mechanisms and further explore the functional regulation of p66Shc in health and disease.

## Abbreviations

AGE: advanced glycation end product
CAD: coronary artery disease
CH1: collagen homology 1
CH2: collagen homology 2
CH2-CB: cytochrome *c* binding domain
EDRF: endothelium derived relaxing factor
EGFR: epidermal growth factor receptor
eNOS: endothelial nitric oxide synthase
FOXO3a: forkhead box protein 3a
HUVEC: human umbilical vein endothelial cell
IGF1: Insulin-like growth factor 1
JNK: c-Jun N-terminal kinase
KLF2: Kruppel-like factor-2
LDL: low-density lipoprotein
MAP: mitogen activated protein
MEF: mouse embryonic fibroblast
mtHSP70: mitochondrial heat shock protein 70
NO: nitric oxide
Nrf2: nuclear factor erythroid 2-related factor 2
o-LDL: oxidized-LDL
PBD: phospho-tyrosine binding domain
PBM: peripheral blood monocyte
PKC: protein kinase C
PTB: phosphotyrosine binding
RAGE: receptor for AGE
ROS: reactive oxygen species
SH2: Src homology 2
SIRT1: Silent information regulator 1
Sos: Son of sevenless
SS: salt-sensitive
Wnt3a: wingless-type MMTV integration site family member 3A

## References

[1] Pawson T. and Gish G.D. (1992) SH2 and SH3 domains: from structure to function. Cell 71, 359–362 10.1016/0092-8674(92)90504-6 1423600

[2] Migliaccio E., Mele S., Salcini A.E., Pelicci G., Lai K.M., Superti-Furga G. (1997) Opposite effects of the p52shc/p46shc and p66shc splicing isoforms on the EGF receptor-MAP kinase-fos signalling pathway. EMBO J. 16, 706–716 10.1093/emboj/16.4.706 9049300 PMC1169672

[3] Migliaccio E., Giorgio M., Mele S., Pelicci G., Reboldi P., Pandolfi P.P. (1999) The p66shc adaptor protein controls oxidative stress response and life span in mammals. Nature 402, 309–313 10.1038/46311 10580504

[4] Ramsey J.J., Tran D., Giorgio M., Griffey S.M., Koehne A., Laing S.T. (2014) The influence of Shc proteins on life span in mice. J. Gerontol. A Biol. Sci. Med. Sci. 69, 1177–1185 10.1093/gerona/glt198 24336818 PMC4172037

[5] Giorgio M., Berry A., Berniakovich I., Poletaeva I., Trinei M., Stendardo M. (2012) The p66Shc knocked out mice are short lived under natural condition. Aging Cell 11, 162–168 10.1111/j.1474-9726.2011.00770.x 22081964

[6] Gertz M. and Steegborn C. (2010) The Lifespan-regulator p66Shc in mitochondria: redox enzyme or redox sensor? Antioxid. Redox Signal. 13, 1417–1428 10.1089/ars.2010.3147 20214499

[7] Pelicci G., Lanfrancone L., Grignani F., McGlade J., Cavallo F., Forni G. (1992) A novel transforming protein (SHC) with an SH2 domain is implicated in mitogenic signal transduction. Cell 70, 93–104 10.1016/0092-8674(92)90536-L 1623525

[8] Ventura A., Luzi L., Pacini S., Baldari C.T. and Pelicci P.G. (2002) The p66Shc longevity gene is silenced through epigenetic modifications of an alternative promoter. J. Biol. Chem. 277, 22370–22376 10.1074/jbc.M200280200 11948181

[9] Kim Y.R., Kim C.S., Naqvi A., Kumar A., Kumar S., Hoffman T.A. (2012) Epigenetic upregulation of p66shc mediates low-density lipoprotein cholesterol-induced endothelial cell dysfunction. Am. J. Physiol. Heart Circ. Physiol. 303, H189–H196 10.1152/ajpheart.01218.2011 22661506 PMC3404699

[10] Trinei M., Giorgio M., Cicalese A., Barozzi S., Ventura A., Migliaccio E. (2002) A p53-p66Shc signalling pathway controls intracellular redox status, levels of oxidation-damaged DNA and oxidative stress-induced apoptosis. Oncogene 21, 3872–3878 10.1038/sj.onc.1205513 12032825

[11] Ihling C., Haendeler J., Menzel G., Hess R.D., Fraedrich G., Schaefer H.E. (1998) Co-expression of p53 and MDM2 in human atherosclerosis: implications for the regulation of cellularity of atherosclerotic lesions. J. Pathol. 185, 303–312 10.1002/(SICI)1096-9896(199807)185:3<303::AID-PATH106>3.0.CO;2-P 9771485

[12] Kim C.S., Jung S.B., Naqvi A., Hoffman T.A., DeRicco J., Yamamori T. (2008) p53 impairs endothelium-dependent vasomotor function through transcriptional upregulation of p66shc. Circ. Res. 103, 1441–1450 10.1161/CIRCRESAHA.108.181644 18988897

[13] Zhou S., Chen H.Z., Wan Y.Z., Zhang Q.J., Wei Y.S., Huang S. (2011) Repression of P66Shc expression by SIRT1 contributes to the prevention of hyperglycemia-induced endothelial dysfunction. Circ. Res. 109, 639–648 10.1161/CIRCRESAHA.111.243592 21778425

[14] Paneni F., Mocharla P., Akhmedov A., Costantino S., Osto E., Volpe M. (2012) Gene silencing of the mitochondrial adaptor p66(Shc) suppresses vascular hyperglycemic memory in diabetes. Circ. Res. 111, 278–289 10.1161/CIRCRESAHA.112.266593 22693349

[15] Costantino S., Paneni F., Virdis A., Hussain S., Mohammed S.A., Capretti G. (2019) Interplay among H3K9-editing enzymes SUV39H1, JMJD2C and SRC-1 drives p66Shc transcription and vascular oxidative stress in obesity. Eur. Heart J. 40, 383–391 10.1093/eurheartj/ehx615 29077881

[16] Bosch-Presegue L., Raurell-Vila H., Marazuela-Duque A., Kane-Goldsmith N., Valle A., Oliver J. (2011) Stabilization of Suv39H1 by SirT1 is part of oxidative stress response and ensures genome protection. Mol. Cell 42, 210–223 10.1016/j.molcel.2011.02.034 21504832

[17] Ray P.D., Huang B.W. and Tsuji Y. (2012) Reactive oxygen species (ROS) homeostasis and redox regulation in cellular signaling. Cell. Signal. 24, 981–990 10.1016/j.cellsig.2012.01.008 22286106 PMC3454471

[18] Du W., Jiang Y., Zheng Z., Zhang Z., Chen N., Ma Z. (2013) Feedback loop between p66(Shc) and Nrf2 promotes lung cancer progression. Cancer Lett. 337, 58–65 10.1016/j.canlet.2013.05.016 23689140

[19] Miyazawa M. and Tsuji Y. (2014) Evidence for a novel antioxidant function and isoform-specific regulation of the human p66Shc gene. Mol. Biol. Cell 25, 2116–2127 10.1091/mbc.e13-11-0666 24807908 PMC4072584

[20] Khanday F.A., Yamamori T., Mattagajasingh I., Zhang Z., Bugayenko A., Naqvi A. (2006) Rac1 leads to phosphorylation-dependent increase in stability of the p66shc adaptor protein: role in Rac1-induced oxidative stress. Mol. Biol. Cell 17, 122–129 10.1091/mbc.e05-06-0570 16251354 PMC1345652

[21] Pinton P., Rimessi A., Marchi S., Orsini F., Migliaccio E., Giorgio M. (2007) Protein kinase C beta and prolyl isomerase 1 regulate mitochondrial effects of the life-span determinant p66Shc. Science 315, 659–663 10.1126/science.1135380 17272725

[22] Shi Y., Cosentino F., Camici G.G., Akhmedov A., Vanhoutte P.M., Tanner F.C. (2011) Oxidized low-density lipoprotein activates p66Shc via lectin-like oxidized low-density lipoprotein receptor-1, protein kinase C-beta, and c-Jun N-terminal kinase kinase in human endothelial cells. Arterioscler. Thromb. Vasc. Biol. 31, 2090–2097 10.1161/ATVBAHA.111.229260 21817106

[23] Arany I., Faisal A., Nagamine Y. and Safirstein R.L. (2008) p66shc inhibits pro-survival epidermal growth factor receptor/ERK signaling during severe oxidative stress in mouse renal proximal tubule cells. J. Biol. Chem. 283, 6110–6117 10.1074/jbc.M708799200 18174162

[24] Orsini F., Migliaccio E., Moroni M., Contursi C., Raker V.A., Piccini D. (2004) The life span determinant p66Shc localizes to mitochondria where it associates with mitochondrial heat shock protein 70 and regulates trans-membrane potential. J. Biol. Chem. 279, 25689–25695 10.1074/jbc.M401844200 15078873

[25] Giorgio M., Migliaccio E., Orsini F., Paolucci D., Moroni M., Contursi C. (2005) Electron transfer between cytochrome c and p66Shc generates reactive oxygen species that trigger mitochondrial apoptosis. Cell 122, 221–233 10.1016/j.cell.2005.05.011 16051147

[26] Khanday F.A., Santhanam L., Kasuno K., Yamamori T., Naqvi A., Dericco J. (2006) Sos-mediated activation of rac1 by p66shc. J. Cell Biol. 172, 817–822 10.1083/jcb.200506001 16520382 PMC2063726

[27] Gertz M., Fischer F., Wolters D. and Steegborn C. (2008) Activation of the lifespan regulator p66Shc through reversible disulfide bond formation. Proc. Natl. Acad. Sci. U.S.A. 105, 5705–5709 10.1073/pnas.0800691105 18413607 PMC2311372

[28] Kumar S., Kim Y.R., Vikram A., Naqvi A., Li Q., Kassan M. (2017) Sirtuin1-regulated lysine acetylation of p66Shc governs diabetes-induced vascular oxidative stress and endothelial dysfunction. Proc. Natl. Acad. Sci. U.S.A. 114, 1714–1719 10.1073/pnas.1614112114 28137876 PMC5321021

[29] Nemoto S. and Finkel T. (2002) Redox regulation of forkhead proteins through a p66shc-dependent signaling pathway. Science 295, 2450–2452 10.1126/science.1069004 11884717

[30] Kumar A., Hoffman T.A., Dericco J., Naqvi A., Jain M.K. and Irani K. (2009) Transcriptional repression of Kruppel like factor-2 by the adaptor protein p66shc. FASEB J. 23, 4344–4352 10.1096/fj.09-138743 19696221 PMC2812051

[31] Chahdi A. and Sorokin A. (2008) Endothelin-1 couples betaPix to p66Shc: role of betaPix in cell proliferation through FOXO3a phosphorylation and p27kip1 down-regulation independently of Akt. Mol. Biol. Cell 19, 2609–2619 10.1091/mbc.e07-05-0424 18385518 PMC2397323

[32] Guo J., Gertsberg Z., Ozgen N. and Steinberg S.F. (2009) p66Shc links alpha1-adrenergic receptors to a reactive oxygen species-dependent AKT-FOXO3A phosphorylation pathway in cardiomyocytes. Circ. Res. 104, 660–669 10.1161/CIRCRESAHA.108.186288 19168439 PMC2861587

[33] Akhmedov A., Montecucco F., Braunersreuther V., Camici G.G., Jakob P., Reiner M.F. (2015) Genetic deletion of the adaptor protein p66Shc increases susceptibility to short-term ischaemic myocardial injury via intracellular salvage pathways. Eur. Heart J. 36, 516a–526a 10.1093/eurheartj/ehu400 25336219

[34] Miller B.S., Blumenthal S.R., Shalygin A., Wright K.D., Staruschenko A., Imig J.D. (2018) Inactivation of p66Shc decreases afferent arteriolar KATP channel activity and decreases renal damage in diabetic Dahl SS rats. Diabetes 67, 2206–2212 10.2337/db18-0308 30131395 PMC6198347

[35] Kumar S., Vikram A., Kim Y.R., Jacobs J S. and Irani K. (2014) P66Shc mediates increased platelet activation and aggregation in hypercholesterolemia. Biochem. Biophys. Res. Commun. 449, 496–501 10.1016/j.bbrc.2014.05.029 24845561

[36] Shahzad K., Gadi I., Nazir S., Al-Dabet M.M., Kohli S., Bock F. (2018) Activated protein C reverses epigenetically sustained p66(Shc) expression in plaque-associated macrophages in diabetes. Commun. Biol. 1, 104 10.1038/s42003-018-0108-5 30271984 PMC6123684

[37] Furchgott R.F. and Zawadzki J.V. (1980) The obligatory role of endothelial cells in the relaxation of arterial smooth muscle by acetylcholine. Nature 288, 373–376 10.1038/288373a0 6253831

[38] Ludmer P.L., Selwyn A.P., Shook T.L., Wayne R.R., Mudge G.H., Alexander R.W. (1986) Paradoxical vasoconstriction induced by acetylcholine in atherosclerotic coronary arteries. N. Engl. J. Med. 315, 1046–1051 10.1056/NEJM198610233151702 3093861

[39] Moritoki H., Hosoki E. and Ishida Y. (1986) Age-related decrease in endothelium-dependent dilator response to histamine in rat mesenteric artery. Eur. J. Pharmacol. 126, 61–67 10.1016/0014-2999(86)90738-7 2875885

[40] Luscher T.F., Tanner F.C., Tschudi M.R. and Noll G. (1993) Endothelial dysfunction in coronary artery disease. Annu. Rev. Med. 44, 395–418 10.1146/annurev.me.44.020193.002143 8476260

[41] Shirasaki Y., Su C., Lee T.J., Kolm P., Cline W.H. Jr and Nickols G.A. (1986) Endothelial modulation of vascular relaxation to nitrovasodilators in aging and hypertension. J. Pharmacol. Exp. Ther. 239, 861–866 3025422

[42] Tschudi M.R., Barton M., Bersinger N.A., Moreau P., Cosentino F., Noll G. (1996) Effect of age on kinetics of nitric oxide release in rat aorta and pulmonary artery. J. Clin. Invest. 98, 899–905 10.1172/JCI118872 8770860 PMC507503

[43] Fleisch J.H. and Spaethe S.M. (1981) Vasodilation and aging evaluated in the isolated perfused rat mesenteric vascular bed: preliminary observations on the vascular pharmacology of dobutamine. J. Cardiovasc. Pharmacol. 3, 187–196 10.1097/00005344-198101000-00017 6160348

[44] Francia P., delli Gatti C., Bachschmid M., Martin-Padura I., Savoia C., Migliaccio E. (2004) Deletion of p66shc gene protects against age-related endothelial dysfunction. Circulation 110, 2889–2895 10.1161/01.CIR.0000147731.24444.4D 15505103

[45] Yamamori T., White A.R., Mattagajasingh I., Khanday F.A., Haile A., Qi B. (2005) P66shc regulates endothelial NO production and endothelium-dependent vasorelaxation: implications for age-associated vascular dysfunction. J. Mol. Cell Cardiol. 39, 992–995 10.1016/j.yjmcc.2005.09.003 16242150

[46] Shi Y., Savarese G., Perrone-Filardi P., Luscher T.F. and Camici G.G. (2014) Enhanced age-dependent cerebrovascular dysfunction is mediated by adaptor protein p66Shc. Int. J. Cardiol. 175, 446–450 10.1016/j.ijcard.2014.06.025 25012499

[47] Pagnin E., Fadini G., de Toni R., Tiengo A., Calo L. and Avogaro A. (2005) Diabetes induces p66shc gene expression in human peripheral blood mononuclear cells: relationship to oxidative stress. J. Clin. Endocrinol. Metab. 90, 1130–1136 10.1210/jc.2004-1283 15562031

[48] Rota M., LeCapitaine N., Hosoda T., Boni A., De Angelis A., Padin-Iruegas M.E. (2006) Diabetes promotes cardiac stem cell aging and heart failure, which are prevented by deletion of the p66shc gene. Circ. Res. 99, 42–52 10.1161/01.RES.0000231289.63468.08 16763167

[49] Menini S., Amadio L., Oddi G., Ricci C., Pesce C., Pugliese F. (2006) Deletion of p66Shc longevity gene protects against experimental diabetic glomerulopathy by preventing diabetes-induced oxidative stress. Diabetes 55, 1642–1650 10.2337/db05-1477 16731826

[50] Camici G.G., Schiavoni M., Francia P., Bachschmid M., Martin-Padura I., Hersberger M. (2007) Genetic deletion of p66(Shc) adaptor protein prevents hyperglycemia-induced endothelial dysfunction and oxidative stress. Proc. Natl. Acad. Sci. U.S.A. 104, 5217–5222 10.1073/pnas.0609656104 17360381 PMC1829289

[51] Alikhani M., Maclellan C.M., Raptis M., Vora S., Trackman P.C. and Graves D.T. (2007) Advanced glycation end products induce apoptosis in fibroblasts through activation of ROS, MAP kinases, and the FOXO1 transcription factor. Am. J. Physiol. Cell Physiol. 292, C850–6 10.1152/ajpcell.00356.2006 17005604

[52] Lu C., He J.C., Cai W., Liu H., Zhu L. and Vlassara H. (2004) Advanced glycation endproduct (AGE) receptor 1 is a negative regulator of the inflammatory response to AGE in mesangial cells. Proc. Natl. Acad. Sci. U.S.A. 101, 11767–11772 10.1073/pnas.0401588101 15289604 PMC511050

[53] Menini S., Iacobini C., Ricci C., Oddi G., Pesce C., Pugliese F. (2007) Ablation of the gene encoding p66Shc protects mice against AGE-induced glomerulopathy by preventing oxidant-dependent tissue injury and further AGE accumulation. Diabetologia 50, 1997–2007 10.1007/s00125-007-0728-7 17611735

[54] Cai W., He J.C., Zhu L., Chen X., Striker G.E. and Vlassara H. (2008) AGE-receptor-1 counteracts cellular oxidant stress induced by AGEs via negative regulation of p66shc-dependent FKHRL1 phosphorylation. Am. J. Physiol. Cell Physiol. 294, C145–C152 10.1152/ajpcell.00350.2007 18032526

[55] Xi G., Shen X., Radhakrishnan Y., Maile L. and Clemmons D. (2010) Hyperglycemia-induced p66shc inhibits insulin-like growth factor I-dependent cell survival via impairment of Src kinase-mediated phosphoinositide-3 kinase/AKT activation in vascular smooth muscle cells. Endocrinology 151, 3611–3623 10.1210/en.2010-0242 20534722 PMC2940520

[56] Paneni F., Volpe M., Luscher T.F. and Cosentino F. (2013) SIRT1, p66(Shc), and Set7/9 in vascular hyperglycemic memory: bringing all the strands together. Diabetes 62, 1800–1807 10.2337/db12-1648 23704521 PMC3661615

[57] Li Q., Kim Y.R., Vikram A., Kumar S., Kassan M., Gabani M. (2016) P66Shc-induced microRNA-34a causes diabetic endothelial dysfunction by downregulating Sirtuin1. Arterioscler. Thromb. Vasc. Biol. 36, 2394–2403 10.1161/ATVBAHA.116.308321 27789474 PMC5293179

[58] Mertens A., Verhamme P., Bielicki J.K., Phillips M.C., Quarck R., Verreth W. (2003) Increased low-density lipoprotein oxidation and impaired high-density lipoprotein antioxidant defense are associated with increased macrophage homing and atherosclerosis in dyslipidemic obese mice: LCAT gene transfer decreases atherosclerosis. Circulation 107, 1640–1646 10.1161/01.CIR.0000056523.08033.9F 12668499

[59] Napoli C., Ackah E., De Nigris F., Del Soldato P., D’Armiento F.P., Crimi E. (2002) Chronic treatment with nitric oxide-releasing aspirin reduces plasma low-density lipoprotein oxidation and oxidative stress, arterial oxidation-specific epitopes, and atherogenesis in hypercholesterolemic mice. Proc. Natl. Acad. Sci. U.S.A. 99, 12467–12470 10.1073/pnas.192244499 12209007 PMC129468

[60] Zeiher A.M., Schachlinger V., Hohnloser S.H., Saurbier B. and Just H. (1994) Coronary atherosclerotic wall thickening and vascular reactivity in humans. Elevated high-density lipoprotein levels ameliorate abnormal vasoconstriction in early atherosclerosis. Circulation 89, 2525–2532 10.1161/01.CIR.89.6.2525 8205660

[61] Lynch S.M., Morrow J.D., Roberts L.J. II and Frei B. (1994) Formation of non-cyclooxygenase-derived prostanoids (F2-isoprostanes) in plasma and low density lipoprotein exposed to oxidative stress *in vitro*. J. Clin. Invest. 93, 998–1004 10.1172/JCI117107 8132786 PMC294019

[62] Keaney J.F. Jr, Shwaery G.T., Xu A., Nicolosi R.J., Loscalzo J., Foxall T.L. (1994) 17 beta-estradiol preserves endothelial vasodilator function and limits low-density lipoprotein oxidation in hypercholesterolemic swine. Circulation 89, 2251–2259 10.1161/01.CIR.89.5.2251 8181150

[63] Diaz M.N., Frei B., Vita J.A. and Keaney J.F. Jr (1997) Antioxidants and atherosclerotic heart disease. N. Engl. J. Med. 337, 408–416 10.1056/NEJM199708073370607 9241131

[64] Napoli C., Martin-Padura I., de Nigris F., Giorgio M., Mansueto G., Somma P. (2003) Deletion of the p66Shc longevity gene reduces systemic and tissue oxidative stress, vascular cell apoptosis, and early atherogenesis in mice fed a high-fat diet. Proc. Natl. Acad. Sci. U.S.A. 100, 2112–2116 10.1073/pnas.0336359100 12571362 PMC149967

[65] Martin-Padura I., de Nigris F., Migliaccio E., Mansueto G., Minardi S., Rienzo M. (2008) p66Shc deletion confers vascular protection in advanced atherosclerosis in hypercholesterolemic apolipoprotein E knockout mice. Endothelium 15, 276–287 10.1080/10623320802487791 19065319

[66] Vikram A., Kim Y.R., Kumar S., Naqvi A., Hoffman T.A., Kumar A. (2014) Canonical Wnt signaling induces vascular endothelial dysfunction via p66Shc-regulated reactive oxygen species. Arterioscler. Thromb. Vasc. Biol. 34, 2301–2309 10.1161/ATVBAHA.114.304338 25147340 PMC6069972

[67] Forstermann U., Xia N. and Li H. (2017) Roles of vascular oxidative stress and nitric oxide in the pathogenesis of atherosclerosis. Circ. Res. 120, 713–735 10.1161/CIRCRESAHA.116.309326 28209797

[68] Shi Y., Luscher T.F. and Camici G.G. (2014) Dual role of endothelial nitric oxide synthase in oxidized LDL-induced, p66Shc-mediated oxidative stress in cultured human endothelial cells. PLoS ONE 9, e107787 10.1371/journal.pone.0107787 25247687 PMC4172699

[69] Noda Y., Yamagishi S., Matsui T., Ueda S., Ueda S., Jinnouchi Y. (2010) The p66shc gene expression in peripheral blood monocytes is increased in patients with coronary artery disease. Clin. Cardiol. 33, 548–552 10.1002/clc.20761 20842738 PMC6653518

[70] Franzeck F.C., Hof D., Spescha R.D., Hasun M., Akhmedov A., Steffel J. (2012) Expression of the aging gene p66Shc is increased in peripheral blood monocytes of patients with acute coronary syndrome but not with stable coronary artery disease. Atherosclerosis 220, 282–286 10.1016/j.atherosclerosis.2011.10.035 22100252

[71] Miao Q., Wang Q., Dong L., Wang Y., Tan Y. and Zhang X. (2015) The expression of p66shc in peripheral blood monocytes is increased in patients with coronary heart disease and correlated with endothelium-dependent vasodilatation. Heart Vessels 30, 451–457 10.1007/s00380-014-0497-4 24676406

[72] Spescha R.D., Glanzmann M., Simic B., Witassek F., Keller S., Akhmedov A. (2014) Adaptor protein p66(Shc) mediates hypertension-associated, cyclic stretch-dependent, endothelial damage. Hypertension 64, 347–353 10.1161/HYPERTENSIONAHA.113.02129 24842918

[73] Miller B., Palygin O., Rufanova V.A., Chong A., Lazar J., Jacob H.J. (2016) p66Shc regulates renal vascular tone in hypertension-induced nephropathy. J. Clin. Invest. 126, 2533–2546 10.1172/JCI75079 27270176 PMC4922697

